# Prevalence and predictors of diabetes mellitus among persons living with HIV: a retrospective cohort study conducted in 4 public healthcare facilities in KwaZulu-Natal

**DOI:** 10.1186/s12889-021-10318-6

**Published:** 2021-02-04

**Authors:** David Mohammed Umar, Panjasaram Naidoo

**Affiliations:** Discipline of Pharmaceutical Sciences, School of Health Sciences, University of KwaZulu-Natal, Westville, Durban, South Africa

**Keywords:** Diabetes, Patient, Factors, HIV, Predictors, Prevalence, PLWHIV

## Abstract

**Background:**

Diabetes mellitus is a chronic non-infectious medical condition which is evident by raised levels of glucose in the blood, because the body cannot produce any or enough of the hormone insulin or use insulin effectively. Diabetes, if not well managed leads to complications such as neuropathy, retinopathy, nephropathy which can be fatal. Some of the factors that predisposes to diabetes include older age, higher body mass index, heredity and hypertension.

With the availability of HAART for managing HIV/AIDS infection, life span of persons living with HIV (PLWHIV) has increased significantly. With increased longevity, the aging population of PLWHIV also face chronic diseases such as diabetes in addition to HIV. The burden of both HIV and diabetes is high in South Africa, particularly in KwaZulu-Natal. Nevertheless, the prevalence of diabetes among PLWHIV in KwaZulu-Natal and its predictors is not well understood. Therefore, this study was conducted to determine the prevalence, predictors of diabetes and the outcome of managing diabetes among PLWHIV.

**Methods:**

This retrospective cohort study was conducted in four public health care facilities in KwaZulu-Natal with a total sample size of 1203 after ethical approval and informed consent were obtained. A pretested questionnaire and hospital patient charts were used to collect data. SPSS version 26 was used to analyze the data using descriptive statistics and logistic regression.

**Results:**

The prevalence of diabetes among PLWHIV was 9%. Just over 47% of those who had diabetes, had uncontrolled blood sugar, with a mean fasting blood sugar (FBS) of 11.7 mmol/L. The predictors of diabetes among PLWHIV were male gender and older age. Male PLWHIV had 65% less chances of having diabetes and those who were between the ages of 18 and 48 years were 88% less probable to have diabetes compared to those who were older than 48 years.

**Conclusion:**

Public sector health care facilities in KwaZulu-Natal need to do much more to manage diabetes in PLWHIV in order to prevent diabetic complications and possible negative impact on the outcome of HIV management.

**Supplementary Information:**

The online version contains supplementary material available at 10.1186/s12889-021-10318-6.

## Introduction

“Diabetes is a group of metabolic diseases characterized by hyperglycemia resulting from defects in insulin secretion, insulin action, or both” [1].

Insulin is an essential hormone produced in the body’s pancreas gland and carries glucose from the bloodstream into the cells of the body where the glucose is transformed into energy. Deficiency of insulin or the cell’s failure to respond to insulin results in hyperglycemia, which is a key feature of diabetes.

If no intervention is done, hyperglycemia can cause damage to different body organs, resulting to the development of debilitating and life-threatening health problems such as cardiovascular disease, neuropathy, nephropathy and eye disease, resulting in retinopathy and blindness. These complications, however, can be slowed down or avoided if diabetes is appropriately managed.

Besides the main types of diabetes, viz. Type 1, type 2 and gestational, there is secondary diabetes which arises as a complication of other diseases like pancreatitis, and hormonal disturbances such as Cushing’s disease [2].

The development of combined antiretroviral therapy has led to the increase in the life span of persons living with HIV (PLWHIV) with treatment, similar to the expected age of the general population [3–5]. With longevity, however, PLWHIV are developing other chronic medical conditions [6–9]. One of these chronic comorbidities is diabetes mellitus.

There are identified risk factors associated with the development of diabetes in PLWHIV that are the same as those in persons without HIV; they include older age, heredity, higher Body Mass Index [BMI], higher triglyceride and hypertension. However, PLWHIV have the additional risk factors of HIV and HIV medicines [10–12]. Antiretroviral medications, such as nucleoside reverse transcriptase inhibitors (NRTI) and protease inhibitors (PI), have been implicated in causing disorders such as insulin resistance, hyperglycaemia and diabetes [11, 13].

This study is particularly important as South Africa is among the highest diabetes prevalent nations in Africa. The convergence of HIV and diabetes in same patients makes it crucial to investigate the extent of the burden and the predictors of diabetes among persons living with HIV, especially as data on the prevalence and the predictors of diabetes in PLWHIV in KwaZulu-Natal is limited.

Understanding the magnitude of the problem and proper management are essential, not only for the prevention of diabetic complications, reduction of mortalities due to the complications or for the improvement in the quality of life but also to prevent possible negative impact on the outcomes of managing HIV. Hence, this study was conducted with the following aim.

## Methods

This was a retrospective cohort study, aimed at determining the prevalence and predictors of diabetes among persons living with HIV (PLWHIV) and assessing the outcome of managing diabetes. The study was conducted in 4 HIV clinics at Public Sector Hospitals in the eThekwini Metro of KwaZulu-Natal (KZN), South Africa with sample size of 281, 286, 345 and 291 respectively, giving a total sample size of 1203. These hospitals were selected based on the different former designated racial settlements. The total of 1203 patients living with HIV that have been on antiretroviral therapy (ART) for at least 6 months, between 2005 and 2019 were randomly selected as follows; letters ‘Y’ and ‘N’ were written on separate folded pieces of paper. Each patient who consented to participate in the study was asked to pick a folded piece of paper. Those who picked ‘Y’ were included in the study, this process was to avoid sampling bias.

The participants had to be 18 years and above, and not pregnant. Those satisfying the criteria were recruited into the study after obtaining their written consent to take part in the study. The following statistical parameters were used to arrive at the minimum sample size of 249 per hospital: Odds ratio = 1.25, type 1 error = 0.05, type 2 error = 0.2 and statistical power = 0.80. Assuming a population variance of 1 and population mean of 0 (normal distribution). A minimum sample size of 996 was determined with a critical Z value = 1.96. Though 996 was required for this study, the number of participants that selected Y was more than the required sample size resulting in a sample size of 1203 which was accepted to allow for dropouts in the study.

Data was collected from October 2018 to October 2019 using both pretested questionnaire and patient chart. The pretesting was done by administering the questionnaires to 4 respondents randomly selected from the 4 hospital where the study was to be done and retrieved after they filled them. The completed questionnaires were checked to determine if the respondents understood the questions as intended by the researcher based on their responses and assessed the time taken to complete the questionnaire so as to avoid designing a questionnaire that was too long that could discourage the respondents from completing it. After that, all necessary adjustments and corrections were made on the questionnaire and made ready for data collection. The sample questionnaires used for pretesting and the participants in the pretesting were excluded in the study.

The questionnaire was designed to obtain information on patient demographics, other information such as diabetes screening at the clinic using fasting blood sugar (FBS), diabetes status, adherence to hypoglycemic medications by the patients, and life-style modification while information on patients management outcomes such as baseline and current CD4 cell counts, baseline and current viral load, initial and current blood sugar were obtained from the hospitals’ patient charts and transcribed into a table designed using Microsoft word. Attached as additional files with this manuscript are blank copies of the questionnaire used to collect data from patients, the patient chart/information sheet into which data was extracted from patients’ hospital records and a completed STROBE checklist.

The statistical package for social sciences (SPSS) software version 26 was used to analyze the data. Descriptive statistics and logistic regression were used in the analyses of data. Missing data in descriptive statistics was accounted for by excluding it from the calculation.

Before the commencement of the study, ethics approval was obtained from the Biomedical Research Ethics Committee of the University of KwaZulu-Natal with the ethics reference number BE314/18. Approvals were also obtained from the KwaZulu-Natal Department of Health and the hospitals where the study was conducted.

Each participant was given the informed consent form from the UKZN Biomedical Research Ethics Committee to read or to be read to and explained thoroughly in either English language or isiZulu according to the preference of the participant. They were given the opportunity to ask questions, the form explained that their participation was voluntary, no incentives or reimbursements for participation in the study and that their anonymity was assured, the purpose for collecting the data was explained and their right to continue or withdraw from the study at any time without any consequence whatsoever was explained to them. Every participant who so consented to participate signed the consent form before he or she was included in the study. Some participants who opted out during the data collection, were excluded from the study.

## Results

A total of 1203 participants were included in this study, with about two third (64%) females while 28 (2.3%) did not indicate their gender. The majority age group was 29–48 years, with about 60% of the participants belonging to this age group. About 33% of the participants had a CD4 count less than 200 cell/μL at the commencement of ART, with a large baseline CD4 count data missing (49.8%) as obtained from patients’ hospital records. Over two third were unemployed as indicated by respondents in the questionnaires. Besides the common opportunistic infections in PLWHIV, other comorbidities present were hypertension (1.0%), epilepsy (0.6%) and renal impairment (0.7%), totally making up 2.3% across the participants (Table 1). Out of the eleven ART regimens prescribed for the patients, TDF/FTC/EFV was the most prescribed regimen (65%) (Table 2). Data in Table 1 and two were extracted from patients’ hospital records and analyzed.

**Table 1:** Demographic information of patients

Variable	Frequency	Percentage (%)
Gender		
Female	770	64.0
Male	405	33.7
Not indicated	28	2.3
Age in years		
18–28	145	12.1
29–48	694	57.7
> 48	313	26.0
Missing data	51	4.2
Baseline CD4		
<200 cells/μL	275	22.9
200–350 cells/μL	156	13.0
351–500 cells/μL	75	6.2
> 500 cells/μL	98	8.1
Missing data	599	49.8
**Employment status**		
Employed	384	31.9
Unemployed	801	66.6
Missing data	18	1.5
Opportunistic infections		
Tuberculosis	90	7.5
Oral candidiasis	6	0.5
**Hepatitis B**	4	0.3
Herpes zoster	5	0.4
Diarrhoea	3	0.2
Septic cellulitis	2	0.2
Pneumonia	2	0.2
Other comorbidities		
Hypertension	12	1.0
Epilepsy	7	0.6
Renal impairment	8	0.7

**Table 2:** Distribution of ART regimens prescribed

ART Regimen	Percentage (%)
ABC/3TC/EFV	13.5
ABC/FTC/EFV	0.1
D4T/3TC/EFV	0.7
TDF/3TC/NVF	0.3
TDF/FTC/ATVr	3.3
TDF/FTC/EFV	65
TDF/FTC/LPVr	4.5
TDF/FTC/NVP	3.1
ZDV/3TC/ATVr	1.9
ZDV/3TC/EFV	0.4
ZDV/3TC/LPVr	7.0

### Prevalence of diabetes mellitus and outcome of diabetes management

**Fig. 1:**
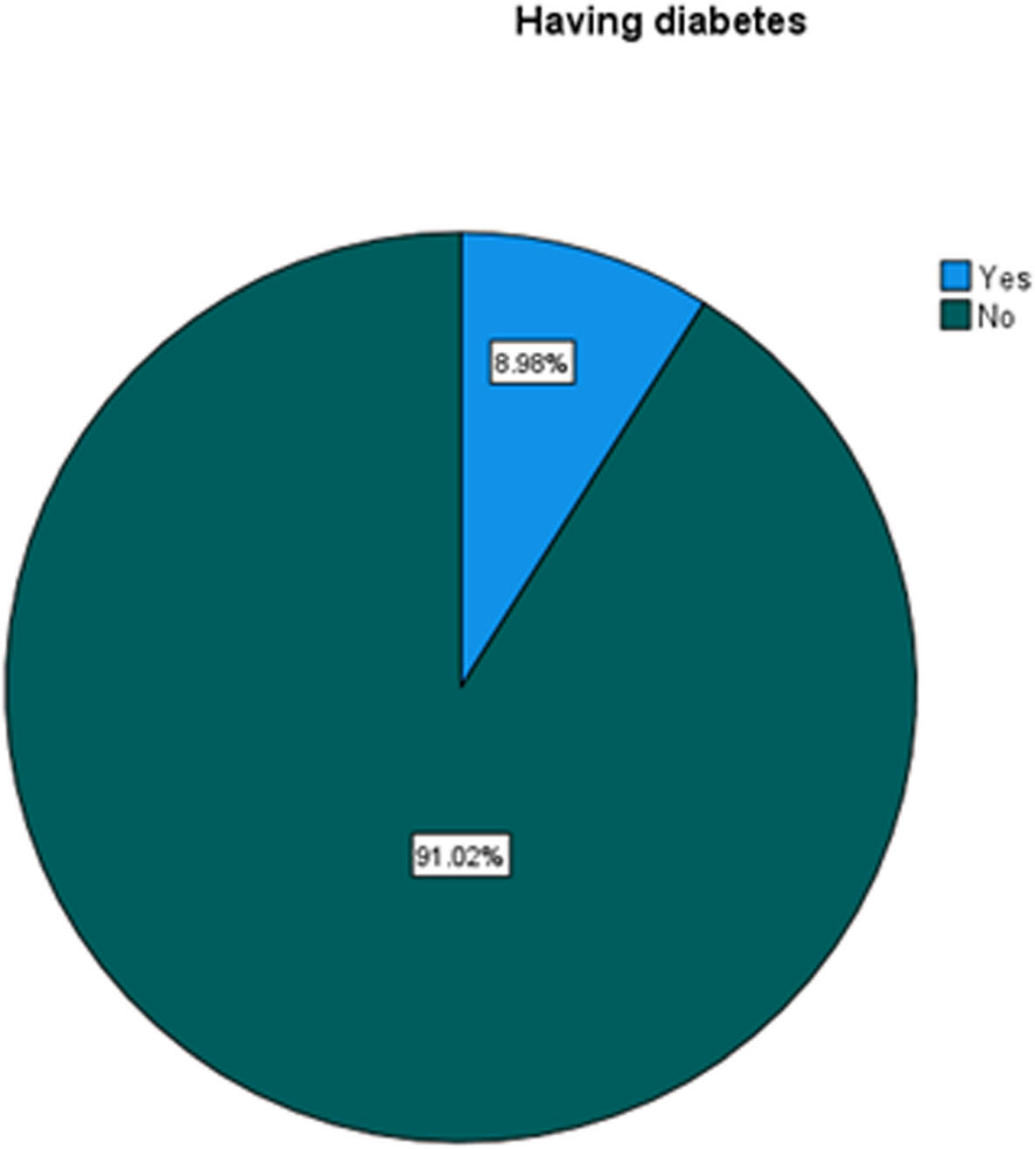
Prevalence of diabetes among persons living with HIV

The prevalence of diabetes among persons living with HIV (PLWHIV) was 9%, (Fig. 1).

Large percentage (61%) of those having diabetes were diagnosed while already on ART.

Almost half (47.1%) of those with diabetes remained with uncontrolled blood sugar (FBS ≥7 mm/L), having a mean FBS of 11.7 mmol/L. Data obtained using questionnaire and patients record, then analyzed.

### Association between patient variables and diabetes mellitus among PLWHIV on ART

Patients’ age and employment status were significantly associated (Cl, 95%) with diabetes among PLWHIV on ART (Table 3).

**Table 3:** Association between patient variables and diabetes among PLWHIV taking ART

Variables	Diabetes, n (%)		Total frequency, n (%)	Chi-square P-value
	No	Yes		
Gender				
Male	363 (92.4)	30 (7.6)	393 (34.6)	0.219
Female	669 (90.2)	73 (9.8)	742 (65.4)	
Age				
18–28	139 (99.3)	1 (0.7)	140 (12.5)	0.000^a^
29–48	643 (95.3)	32 (4.7)	675 (60.5)	
> 48	233 (77.4)	68 (22.6)	301 (27.0)	
Level of Education				
No formal education	40 (87.0)	6 (13.6)	46 (4.2)	0.109
Primary	175 (87.1)	26 (12.9)	201 (18.5)	
High school	601 (92.2)	51 (7.8)	652 (60.0)	
Tertiary	173 (92.0)	15 (8.0)	188 (17.3)	
Employment Status				
Employed	349 (93.6)	24 (6.4)	373 (32.6)	0.030^a^
unemployed	692 (89.6)	80 (10.4)	772 (67.4)	
Alcohol consumption				
Yes	189 (92.2)	16 (7.8%)	205 (18.1)	0.505
No	841 (90.7)	86 (9.3)	927 (81.9)	
Initial CD4 count (cells/mm3)				
< 200	234 (88.6)	30 (11.4)	264 (44.9)	0.414
200–350	142 (91.0)	14 (9.0)	156 (26.5)	
351–500	68 (93.2)	5 (6.8)	73 (12.4)	
> 500	89 (93.7)	6 (6.3)	95 (16.2)	
Current CD4 count (cells/mm3)				
< 200	50 (94.3)	3 (5.7)	53 (8.3)	0.386
200–350	99 (93.4)	7 (6.6)	106 (16.7)	
351–500	133 (90.5)	14 (9.5)	147 (23.1)	
> 500	293 (88.8)	37 (11.2)	330 (51.9)	
Initial viral load (copies/mm3)				
High (≥100,000)	22 (95.7)	1 (4.3)	23 (14.6)	0.137
Low (10,000 – 99,000)	19 (79.2)	5 (20.8)	24 (15.3)	
Lower (< 10,000)	100 (90.9)	10 (9.1)	110 (70.1)	
Current viral load (cells/mm3)				
‘Detectable’	340 (90.9)	34 (9.1)	374 (59.0)	0.587
LTDL	233 (89.6)	27 (10.4)	260 (41.0)	

Key: ^a^ = Statistically significant

### Predictors of diabetes among PLWHIV on ART

In a stepwise forward likelihood ratio multivariate logistic regression model, female gender and age were the predictors of diabetes in PLWHIV on ART (Table 4).

**Table 4:** Predictors of diabetes in PLWHIV on ART (Multi-covariate and uni-covariate logistic regression)

Variables	COR (95%CI)	COR *P*-Value	aOR(95%CI)	aOR P-Value
Gender				
Male	0.76 (0.49–1.18)	0.220	0.35 (0.15–0.82)	0.016*
Female	1		1	
Age				
18–48	0.14 (0.09–0.22)	0.000*	0.12 (0.06–0.26)	0.000*
> 48	1		1	
Duration on ART				
			1 (0.99–1.01)	0.473
Level of education				
No formal education	1.73 (0.63–4.74)	0.286		
Primary	1.71 (0.88–3.35)	0.115		
High school	0.98 (0.54–1.78)	0.944		
Tertiary	1			
Employment Status				
Employed	0.60 (0.37–0.96)	0.032*		
unemployed	1			
Alcohol consumption				
Yes	0.83 (0.48–1.44)	0.506		
No	1			
Baseline CD4 cells count				
> 200 cells/μL			1.90 (0.91–3.98)	0.088
≤ 200 cells/μL			1	
Current CD4 cells count				
> 200 cells/μL			1.04 (0.25–4.32)	0.957
≤ 200 cells/μL			1	

Keys: 1 = the reference category; *COR* Crude Odd Ratio, *CI* Confidence interval, *aOR* Adjusted Odd Ratio (Logistic regression),  * = statistically signficant

The probability for diabetes mellitus in male PLWHIV on ART was 65% less than that of females (aOR = 0.35, 95% Cl = 0.15–0.82, *P*-value = 0.016) (Table 4).

The likelihood of diabetes mellitus in PLWHIV on ART who were between the ages 18 and 48 years was 88% less than those that were older than 48 years. (aOR = 0.12, 95% Cl = 0.06–0.26, P-value = 0.000) (Table 4).

As can be seen from Table 3 above, there was statistically significant association between the age and employment status of PLWHIV and having diabetes, at 95% confidence level.

## Discussion

In this study 9% of the participants living with HIV (PLWHIV) had diabetes (Fig. 1). South Africa, where the study was conducted has a high HIV prevalence as 20.4% of adults between the ages of 15 and 49 live with HIV [14]. In addition, the prevalence of diabetes among South Africa’s adult general population was 5.4% [15]., yet the prevalence of diabetes among PLWHIV was much higher at 9%. As shown in this study.

This high prevalence of diabetes among PLWHIV as shown in our study, is consistent with findings by some other earlier studies [12, 16–18]. However, a study by Diabetes Focus eMag [19] indicated that prevalence of diabetes among PLWHIV is similar to that among the general population. This difference in findings by different studies may be due to differences in the prevalence of diabetes amongst different populations, or differences in participant’s lifestyles.

Another finding from this study relating to gender has shown that the prevalence of diabetes among females PLWHIV was higher (9.5%) than that of males (7.4%). This finding is similar with a study by Hernandez-Ronieu et al., where in 2017 [20], it was shown that the prevalence of diabetes among females living with HIV was higher than that of males living with HIV. However, the same study showed that the prevalence of diabetes is higher in males among the general population. Furthermore, in this South African study it was found that female gender is a predictor for diabetes in PLWHIV, as males living with HIV were 65% less likely to have diabetes than females (Table 4). This finding was similar with other studies which indicated that female who are HIV positive are more likely to have non-communicable diseases (NDC) co-morbidity [21, 22]. Hence, females living with HIV should be screened for diabetes repeatedly at close interval, in other to detect diabetes early and manage them accordingly.

Though this study found that 61% of the PLWHIV were diagnosed with diabetes after the commencement of antiretroviral therapy, there was no significant association found between when ART was commenced and the incidence of diabetes mellitus. Earlier studies vary in their findings with regards to the association between ART and diabetes, with some studies showing similar results to this study [19], while other studies were contrary to the findings of this study, in that, they showed association between ART and diabetes [16–18, 23]. People who test positive for HIV should be tested for diabetes before the commencement of ART and periodically thereafter.

Almost half (47.1%) of the PLWHIV with diabetes in this study remained with uncontrolled blood sugar (FBS ≥7 mm/L, with Mean FBS of 11.7 mmol/L), this is particularly of concern, as this predisposes them to diabetic complications such as retinopathy, neuropathy, nephropathy among others. These complications, if allowed to occur will further increase the disease burden and pill burden for this group of patients. Therefore, this study further sheds light on this issue to help clinicians understand the burden of diabetes among PLWHIV and appreciate the possible impact of uncontrolled blood sugar among these patients, with a view to mitigating the impact of the convergence of these chronic conditions by integrating HIV care and the care of non-communicable chronic diseases such as diabetes.

This study also showed that older age is a predictor to diabetes in PLWHIV, such that the likelihood of diabetes for those older than 48 years of age was 88% compared to those that are younger than 48 years of age (Table 4). This is similar with other studies which showed that old age is a risk factor to chronic comorbidities in PLWHIV [21, 22]. As ART increases the life span of PLWHIV, predisposing them to chronic medical conditions such as diabetes, clinicians should give adequate attention to diabetes in PLWHIV as they do to other comorbidities.

However, the current (at the time of the study) blood sugar measurement for some of the patients with diabetes were missing, this might have affected the level of accuracy of the mean fasting blood sugar found in this study (11.7 mmol/L). Although 39% of the participants were diagnosed with diabetes before the commencement of ART, there was a limitation of data for the duration of diabetes in most of the participants (61%) who were diagnosed with diabetes after the commencement of ART.

## Conclusion/recommendations

In KwaZulu-Natal, the prevalence of HIV among PLWHIV (9%), the prevalence among females was higher (9.5%) than that of males (7.4%) and predictors of diabetes among PLWHIV were female gender and older age. About half (47.1%) of the people with diabetes had uncontrolled blood sugar with a mean FBS of 11.7 mm/L. There was no association between ART and diabetes. It is important to integrate the detection and the care of non-communicable chronic diseases including diabetes into the existing HIV management structure, as this has the potential to maximize the use of available resources as well as improving the outcomes of management of diseases such as diabetes. Regular and continuous testing for diabetes should be carried out and those found to be diabetic should be adequately managed to prevent diabetic complications as well as prevent possible interference with the outcomes of managing HIV.

## Supplementary Information

**Additional file 1.** Questionnaire. This was used to collect data from patients which includes demographics, understanding of their medical conditions, adherence to medications, perceptions about treatments among others.

**Additional file 2.** Patient information sheet. This was used to obtain patient information from patients’ hospital records such as diagnosis, date of diagnosis, treatments/management, outcomes of treatments among others.

## Data Availability

The datasets used and/or analyzed during the current study are available from the corresponding author on reasonable request.

## Abbreviations

AIDS: Acquired Immunodeficiency Syndrome
ART: Antiretroviral Therapy
BMI: Body Mass Index
BREC: Biomedical Research Ethics Committee
FBS: Fasting Blood Sugar
HAART: Highly Active Antiretroviral Therapy
HIV: Human Immunodeficiency Virus
KZN: KwaZulu-Natal
NRTI: Nucleoside Reverse Transcriptase Inhibitors
PI: Protease Inhibitors
PLWHIV: Persons Living With HIV
SPSS: Statistical Package for Social Sciences
UKZN: University of KwaZulu-Natal
