# Supplementary material for: Prevalence and predictors of diabetes mellitus among persons living with HIV: a retrospective cohort study conducted in 4 public healthcare facilities in KwaZulu-Natal
Source: BMC Public Health. 2021 Feb 4;21:288. doi: 10.1186/s12889-021-10318-6 (PMC7863241; doi:10.1186/s12889-021-10318-6)
Supplement: Supplementary file 1 — Additional file 1. Questionnaire. This was used to collect data from patients which includes demographics, understanding of their medical conditions, adherence to medications, perceptions about treatments among others. [file 12889_2021_10318_MOESM1_ESM.docx]

**Dear Participant,**

**Thank you for agreeing to participate in this study.**

**You indicated you are taking medication for your HIV. Some individuals have identified many issues concerning their medicine-taking behavior and we are interested in your experiences. There is no right or wrong answer. Please answer each question based on your personal experience with your HIV medication.**

**Please TICK the appropriate box**

1. Gender

| Male | Female | Transgender |
| --- | --- | --- |
|  |  |  |

1. How old are you (In years)?

| 18-23 | 24-28 | 29-33 | | 34-38 | 39-43 | | 44-48 | 49-53 | | 54-58 | 59-63 | | 64-68 | Above 68 Years |
| --- | --- | --- | --- | --- | --- | --- | --- | --- | --- | --- | --- | --- | --- | --- |
|  |  |  |  | |  |  | |  |  | |  |  | |  |

1. What is your educational level?

| Primary school | High school | Tertiary level | No formal education |
| --- | --- | --- | --- |
|  |  |  |  |

1. Are you employed?

| Yes | No |
| --- | --- |
|  |  |

1. When do you usually take your HIV medicine (s) every day?

| In the morning | In the afternoon | In the evening | At night |
| --- | --- | --- | --- |
|  |  |  |  |

1. Do you sometimes forget to take your medicine for HIV?

| Yes | No |
| --- | --- |
|  |  |

1. Thinking over the past 2 weeks, were there any days when you did not take your HIV medicine(s)?

| Yes | No |
| --- | --- |
|  |  |

1. If yes, what was the reason(s) for not taking your HIV medicine(s)?

| I forgot | Too many people around | I got tired of taking it | Was too busy | Others (Please specify) |
| --- | --- | --- | --- | --- |
|  |  |  |  |  |

1. Thinking over the past 1 month, were there any days when you did not take your HIV medicine(s)?

| Yes | No |
| --- | --- |
|  |  |

1. If yes what was the reason(s) for not taking your HIV medicine(s)?

| I forgot | Too many people around | I got tired of taking it | Was too busy | Others (Please specify) |
| --- | --- | --- | --- | --- |
|  |  |  |  |  |

1. Have you ever cut back or stopped taking your HIV medicine(s) without telling your doctor/nurse because you felt worse when you took it?

| Yes | No |
| --- | --- |
|  |  |

1. What do you mean by feeling worse?

| Felt nauseous | Had diarrhea | Had Headache | Others (Please specify) |
| --- | --- | --- | --- |
|  |  |  |  |

1. Did you visit the doctor/nurse thereafter?

| Yes | No |
| --- | --- |
|  |  |

1. If Yes, what did the doctor/nurse do?

| Treated me but did not change the medicines | Treated me and changed the medicines | Others (Please specify) |
| --- | --- | --- |
|  |  |  |

1. When you travel or leave home, do you sometimes forget to take along your HIV medicine(s)?

| Yes | No |
| --- | --- |
|  |  |

1. Did you take all your HIV medicines yesterday?

| Yes | No |
| --- | --- |
|  |  |

1. When you feel like your symptoms are under control, do you sometimes stop taking your HIV medicine(s)?

| Yes | No |
| --- | --- |
|  |  |

1. If yes, when do you start taking your medicines again?

| The next day | After 2 days | After 5 days | After 1 week | After 1 month | Others (Please specify) |
| --- | --- | --- | --- | --- | --- |
|  |  |  |  |  |  |

1. Taking HIV medicine(s) every day is a real inconvenience for some people. Do you ever feel inconvenienced about sticking to your treatment plan?

| Yes | No |
| --- | --- |
|  |  |

1. How often do you have difficulty remembering to take all your HIV medicines?

| Never | Once in a while | Sometimes | Usually | All the time |
| --- | --- | --- | --- | --- |
|  |  |  |  |  |

1. Do you take alcohol?

| Yes | No |
| --- | --- |
|  |  |

1. If yes, how often do you take alcohol?

| Occasionally | Sometimes | Usually | Everyday |
| --- | --- | --- | --- |
|  |  |  |  |

1. When do you usually take alcohol?

| In the morning | In the afternoon | In the evening | Anytime |
| --- | --- | --- | --- |
|  |  |  |  |

1. Do you take herbal/traditional medicines?

| Yes | No |
| --- | --- |
|  |  |

1. If yes, have you told your doctor/nurse that you take herbal/traditional medicine?

| Yes | No |
| --- | --- |
|  |  |

1. If no, why have you not told the doctor/nurse?

| Afraid to tell them | Did not think it necessary to tell them | My traditional healer asked me not to tell the doctor or nurse. | Others [please specify] |
| --- | --- | --- | --- |
|  |  |  |  |

1. Do you take your herbal/traditional medicines and your HIV medicines at the same time?

| Yes | No |
| --- | --- |
|  |  |

1. Do you take supplements?

| Yes | No |
| --- | --- |
|  |  |

1. Do you take your supplements and your HIV medicines at the same time?

| Yes | No |
| --- | --- |
|  |  |

1. Do you think it is necessary you take your HIV medicines every day?

| Yes | No |
| --- | --- |
|  |  |

1. Do you think the HIV medicines you take can really keep you healthy?

| Yes | No |
| --- | --- |
|  |  |

1. Did you ever stop taking your HIV medicines after taking it for a long time, like 2 to 3 years?

| Yes | No |
| --- | --- |
|  |  |

1. Did you tell any of your family members your HIV status?

| Yes | No |
| --- | --- |
|  |  |

1. If yes, do your family members encourage you to always take your HIV medicines?

| Yes | No |
| --- | --- |
|  |  |

1. Whenever you fall sick do you receive enough care and assistance from your family?

| Yes | No |
| --- | --- |
|  |  |

1. Do you get financial assistance from your family when you need it to transport yourself to the clinic?

| Yes | No |
| --- | --- |
|  |  |

1. Do you experience any form of discrimination from your family or friends?

| Yes | No |
| --- | --- |
|  |  |

1. If your answer to the above question is yes, please specify the kind of discrimination you experience?

| They don’t share cutleries with me | They don’t like relating with me | Others (Please specify) |
| --- | --- | --- |
|  |  |  |

1. Do you have diabetes?

| Yes | No |
| --- | --- |
|  |  |

1. When were you diagnosed with diabetes?

| Before I was diagnosed with HIV | About the same time I was diagnosed with HIV | About 6 months after I started taking medicines for HIV | Longer than 6 months after I started taking medicines for HIV |
| --- | --- | --- | --- |
|  |  |  |  |

1. Are you receiving medicines for diabetes?

| Yes | No |
| --- | --- |
|  |  |

1. Are you receiving your medicines for diabetes in this hospital?

| Yes | No |
| --- | --- |
|  |  |

1. After starting your diabetic treatment do you feel better than before?

| Yes | No |
| --- | --- |
|  |  |

1. After starting your diabetic treatment do you feel worse than before?

| Yes | No |
| --- | --- |
|  |  |

1. Do you get your medicines for diabetes from this hospital?

| Yes | No |
| --- | --- |
|  |  |

1. Do you sometimes not take your medicines for diabetes?

| Yes | No |
| --- | --- |
|  |  |

1. Do they usually check your blood sugar in the hospital?

| Yes | No |
| --- | --- |
|  |  |

1. How often do the check your blood sugar in the hospital?

| Every visit | After some visit | Rarely | Never |
| --- | --- | --- | --- |
|  |  |  |  |

1. When they check your blood sugar how often is your blood sugar high?

| Never | Rarely | Sometimes | Most times | Always | I don’t know |
| --- | --- | --- | --- | --- | --- |
|  |  |  |  |  |  |

1. Are there some food or drinks you were asked not to eat or drink much?

| Yes | No |
| --- | --- |
|  |  |

1. If your answer to the above question (number 50) is YES, do you obey the instruction?

| Yes | No |
| --- | --- |
|  |  |
