# Supplementary material for: Prevalence and predictors of diabetes mellitus among persons living with HIV: a retrospective cohort study conducted in 4 public healthcare facilities in KwaZulu-Natal
Source: BMC Public Health. 2021 Feb 4;21:288. doi: 10.1186/s12889-021-10318-6 (PMC7863241; doi:10.1186/s12889-021-10318-6)
Supplement: Supplementary file 2 — Additional file 2. Patient information sheet. This was used to obtain patient information from patients’ hospital records such as diagnosis, date of diagnosis, treatments/management, outcomes of treatments among others. [file 12889_2021_10318_MOESM2_ESM.docx]

**PATIENT INFORMATION SHEET**

| S/N | Doctor’s Code | | Patient Code | | Sex | | Age | | Date ARVs Started | Date of Last Visit | | | Initial CD4 Count | Current CD4 Count | | Initial Viral Load | | Current Viral Load | | Opportunistic Infections | | Other Comorbidities | Initial Weight (Kg) | | Current Weight (Kg) | |
| --- | --- | --- | --- | --- | --- | --- | --- | --- | --- | --- | --- | --- | --- | --- | --- | --- | --- | --- | --- | --- | --- | --- | --- | --- | --- | --- |
|  |  | |  | |  | |  | |  |  | | |  |  | |  | |  | |  | |  |  | |  | |
| Initial Clinical Stage | | Current Clinical Stage | | ARV Regimen | | Date | | Changes in ARV Regimen | | | Date | Reasons for the Changes | | | Initial Blood Sugar Level (mMol/L) | | Date | | Current Blood Sugar Level (mMol/L) | | Date | Diabetic Complications | | Diabetes Medications | |  |
|  | |  | |  | |  | |  | | |  |  | | |  | |  | |  | |  |  | |  | |  |
